# An Update Report on the Biosafety and Potential Toxicity of Fullerene-Based Nanomaterials toward Aquatic Animals

**DOI:** 10.1155/2021/7995223

**Published:** 2021-07-17

**Authors:** Nemi Malhotra, Gilbert Audira, Agnes L. Castillo, Petrus Siregar, Johnsy Margotte S. Ruallo, Marri Jmelou Roldan, Jung-Ren Chen, Jiann-Shing Lee, Tzong-Rong Ger, Chung-Der Hsiao

**Affiliations:** ^1^Department of Biomedical Engineering, Chung Yuan Christian University, Chung-Li 320314, Taiwan; ^2^Department of Chemistry, Chung Yuan Christian University, Chung-Li 320314, Taiwan; ^3^Department of Bioscience Technology, Chung Yuan Christian University, Chung-Li 320314, Taiwan; ^4^Faculty of Pharmacy, The Graduate School and Research Center for the Natural and Applied Sciences, University of Santo Tomas, Manila 1008, Philippines; ^5^The Graduate School, University of Santo Tomas, Manila 1015, Philippines; ^6^Faculty of Pharmacy and Graduate School, University of Santo Tomas, Manila 1008, Philippines; ^7^Department of Biological Science & Technology, College of Medicine, I-Shou University, Kaohsiung 82445, Taiwan; ^8^Department of Applied Physics, National Pingtung University, Pingtung 900391, Taiwan; ^9^Center for Nanotechnology, Chung Yuan Christian University, Chung-Li 320314, Taiwan; ^10^Research Center for Aquatic Toxicology and Pharmacology, Chung Yuan Christian University, Chung-Li 320314, Taiwan

## Abstract

Fullerene molecules are composed of carbon in the form of a hollow sphere, tube, or ellipsoid. Since their discovery in 1985, they have gained a lot of attention in many science fields. The unique carbon cage structure of fullerene provides immense scope for derivatization, rendering potential for various industrial applications. Thus, the prospective applications of fullerenes have led to assorted fullerene derivatives. In addition, their unique chemical structure also eases them to be synthesized through various kinds of conjugating techniques, where fullerene can be located either on the backbone or the branch chain. In this review, we have compiled the toxicity and biosafety aspects of fullerene in aquatic organisms since the frequent use of fullerene is likely to come in contact and interact with the aquatic environment and aquatic organisms. According to the current understanding, waterborne exposure to fullerene-based nanomaterials indeed triggers toxicities at cellular, organic, molecular, and neurobehavioral levels.

## 1. Introduction and Application of Fullerene

### 1.1. Introduction of Fullerene

Carbon is known to be found in two allotropes, namely, diamond and graphite. In 1985, Kroto, Curl, and Smalley discovered another allotropic form of carbon, which was fullerene. Because of the discovery and their pioneering efforts, they received the Nobel Prize in 1996 [1]. Fullerenes are regarded as three-dimensional analogs of benzene and composed of carbon atoms that are joined by single and double bonds. Together with fused rings of five to seven carbon atoms, these bonds form a closed or partially closed mesh. Carbon atoms in a fullerene molecule can be found in a variety of sizes and shapes, including hollow spheres, ellipsoids, and tubes [2, 3]. The discovered fullerenes consist of various *n* numbers of carbon atoms, which obeyed a specific rule.

C_60_, one of the most well-known and abundant forms of fullerenes, is composed of *n* = 60 carbon atoms, which are arranged in a spherical cage structure of about 7 Å in diameter. Furthermore, to honor the inventor of geodesic domes in the 1960s, it also goes by the name of buckminsterfullerene [2]. Informally, C_60_ fullerenes are also known as buckyballs since their shape resembles a soccer ball shape. In C_60_ fullerenes, there are two types of C-C bonds with distinct lengths: C_5_-C_5_ single bonds in the pentagons and C_5_-C_6_ double bonds in the hexagons with 1.45 Å and 1.40 Å in distance, respectively [4]. The C_60_ has a low specific gravity relative to the diamond (1.65 compared to 3.51). Chemically, the molecule is very stable owing to the fact that destruction of the cages requires temperatures above 1000°C [4, 5]. In addition, besides C_60_, other fullerenes can consist of numerous carbon atoms, ranging from 30 to 980, creating various structures with different characteristics and applications.

### 1.2. Applications of Fullerene

**Figure 1:**
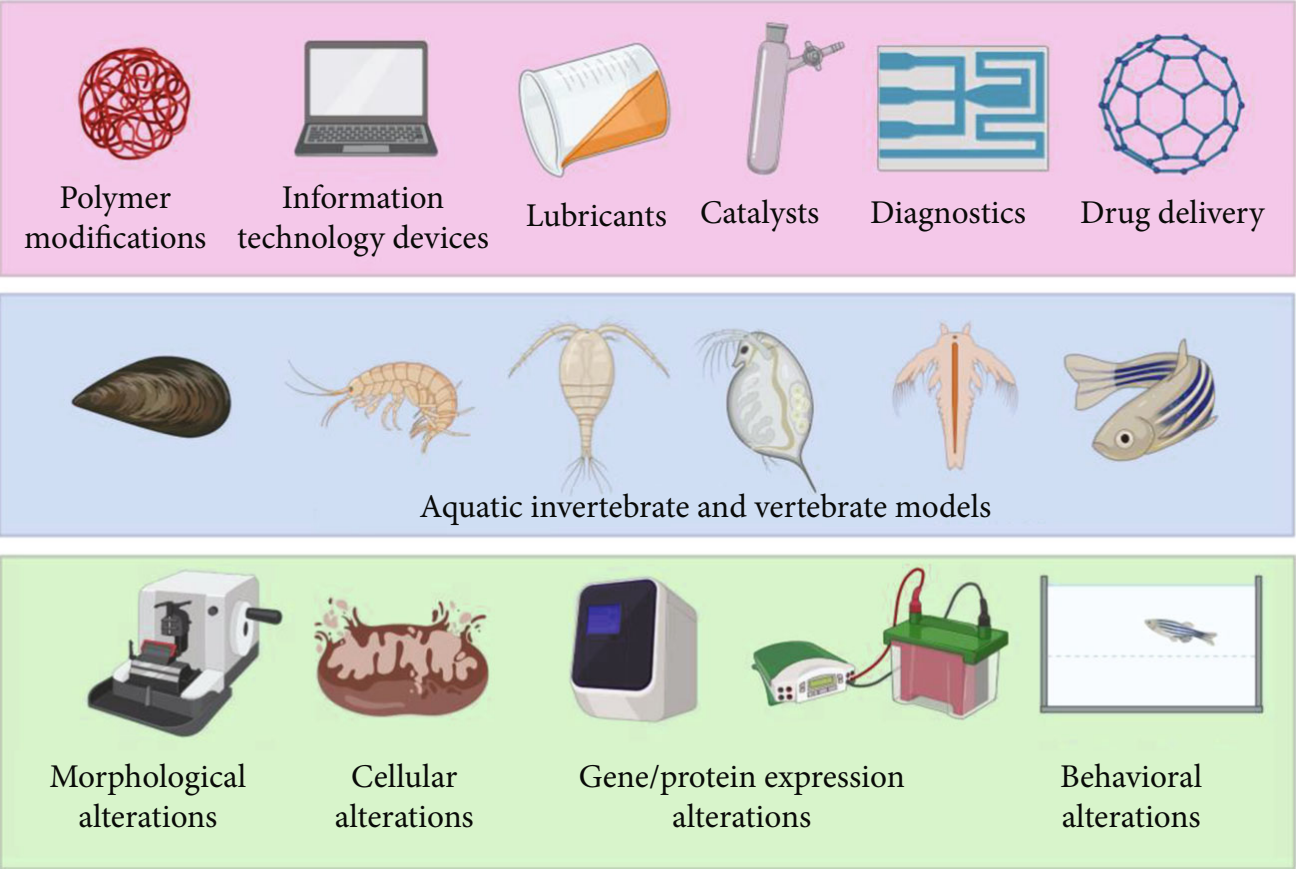
Summary of applications, animal models, and methods of fullerene toxicity assessment in aquatic species. The industrial and biomedical applications of fullerene were compiled in the upper panel (pink color). The invertebrate and vertebrate animal models used to perform fullerene toxicity assessment were compiled in the middle panel (blue color). The various methods used to detect fullerene-induced changes at either morphological, cellular, gene/protein expressional, or behavioral levels were summarized in the bottom panel (green color).

These new forms of carbon's distinctive physical and chemical properties have taken numerous scientists to propose several technological applications. Nowadays, C_60_ molecules have attracted a lot of attention since they have a high electron affinity and antioxidant and radical scavenging properties, which can absorb many free radicals responsible for aging the skins and grafting surfactants or hydrophilic polymers in aqueous environments [6–8]. Fullerenes can be integrated into shafts and frames for strengthening composite materials with very thin-walled, lightweight, robust carbon structures that make them currently be applied in cosmetic products and sporting goods industries [9]. Recently, several investigations suggested that a lot of the proposed fullerenes are applicable in many areas, including information technology devices, lubricants, catalysts, diagnostics, pharmaceuticals, polymer modifications, energy applications, and environmental fields [2, 3, 6, 10]. Furthermore, the unique cage structure of fullerenes, coupled with their immense scope for derivatization, opens up the potential of fullerenes to be a therapeutic agent. In addition, it has also been extensively used in many biomedical applications, including MRI contrast agents, X-ray imaging contrast agents, anti-HIV drugs, targeted drug delivery systems, and photodynamic therapy [11–13]. Therefore, the production and usage of fullerenes are expected to escalate in the future. However, most fullerenes are nonbiodegradable molecules whose potential toxicity has not been thoroughly investigated so far. The increased demand for fullerenes and their mass production have raised biosafety and environmental concerns. *In vivo* toxicity testing provides intact systems for the prediction of biological responses. Aquatic invertebrates and vertebrates provide cost, labor, and time-effective platforms for these studies. These *in vivo* study models provide results on many cellular, anatomical, and physiological characteristics. Moreover, their small size, speedy development, and short life cycle make them attractive for evaluating nanomaterial's toxicological effects. Various methods of toxicity assessments have been developed at different levels of these *in vivo* model systems, such as morphological alterations assessed under a microscope, cellular alterations, gene/protein expression alteration, biochemical analysis, and behavior alterations. The applications of fullerene, aquatic invertebrate, and vertebrate model systems used to assess the toxicity and methods of toxicity analysis of fullerene are compiled in Figure 1.

## 2. Types and Chemical Composition of Fullerene

### 2.1. Types of Fullerenes

Fullerenes occur in nature and have a similar structure to graphite, except that they may contain pentagonal rings [14]. Small quantities of fullerene in the form of C_60_, C_70_, C_76_, and C_84_ might be found hidden in carbon soot [15]. In 2010, by using Spitzer infrared telescope, NASA also found C_60_ and C_70_ in a cloud of cosmic dust surrounding a star [16]. Generally, the synthesis of these fullerenes starts by forming fullerene-rich soot. The original process generates an electric arc between two graphite rods in an inert atmosphere resulting in a vaporized carbon that is subsequently cooled into sooty residue [1, 17]. In addition, laser ablation of graphite targets or laser pyrolysis of aromatic hydrocarbons can also form carbon soot [17]. By contrast, combustion is the most effective method to produce commercial fullerenes in high-temperature, low-pressure premixed flat flames [18]. Later, through these chemical processes, a solid mixture of various fullerenes and other carbons is formed. Afterward, by using suitable organic solvents, small amounts of fullerenes are extracted from the soot and separated by liquid chromatography [17].

The closed buckyballs and cylindrical carbon nanotubes are two major fullerene classes with distinct properties and applications, although some hybrid structures also exist besides these families, including carbon nanobuds. These classes have unique properties due to their carbon atom arrangement. Each carbon atom in the closed fullerenes, especially C_60_, bonds to three others and is sp^2^ hybridized. These delocalized electrons on the surface of a three-dimensional structure stabilize the spheroid structure of C_60_ by resonance [19]. Distorted buckyballs with *n* = 24, 28, 32, 36, and 50 were also obtained, but they are predicted to be unstable. Other relatively common clusters with *n* = 70, 72, 74, 76, 80, 82, and 84 exist but are less abundant in the experimentally produced carbon soot [20]. A general rule is observed that the chemical reactivity significantly decreases with the increasing size of the fullerene molecule. The closed buckyballs, in contrast to graphite, are not electrically conductive. Due to their spherical shape, buckyballs are well known as suitable lubricants. Moreover, buckyballs might be useful in medicine deliveries in the future because of their unique and hollow cage. On the other hand, cylindrical fullerenes, another major family of fullerenes, are known as carbon nanotubes or buckytubes. Every nanotube is a single molecule consisting of millions of carbon atoms. Generally, this molecule's width is only a few nanometers; however, the length ranges from less than a micrometer to several millimeters [21]. Most of the time, the nanotubes have closed ends; however, it is also possible to be open-ended. Carbon nanotubes exhibit higher tensile strength, flexibility, elasticity, and high thermal conductivity [22, 23]. They are often utilized to reinforce composite materials with improved mechanical, electrical, and thermal properties. In addition, these nanotubes may act electrically as either a metal or a semiconductor depending on the hexagonal units' orientation in the tube wall with the tube axis [24].

### 2.2. Chemical Composition of Fullerene

Since every fullerene possesses abundant cyclohexanes, they are very aromatic and have stable and inert carbon bonds. The insolubility in aqueous media and poor miscibility of fullerenes limit the biological applications, and its strong tendency to form self-aggregate also leads to phase separation problems [25, 26]. Since pristine fullerenes and carbon nanotubes lack hydrogen atoms or other groups on their surface, they cannot undergo substitution reactions. Because of this reason, they need to carry out surface modification in order to promote the functionalization on their surface [26]. It is worth noting that fullerenes are the only known allotropic carbons that are soluble in common organic solvents (for example, toluene) despite a limited solubility in most solvents [4, 26–28]. Once fullerenes are dissolved in organic solvents, various chemical reactions tend to proceed in solution, and thus, numerous fullerene derivatives are formed. In producing these derivatives, fullerenes can undergo various chemical reactions, e.g., oxidation, reduction, nucleophilic substitutions, halogenations, hydrogenations, radical additions, transition-metal-complex formations, and regioselective functionalization reactions [2]. By attaching active groups to their surface and modifying their basic properties to be adjusted to specific functions, the increment in fullerenes reactivity has been demonstrated.

Functionalized buckyballs are primarily divided into two classes: endohedral and exohedral fullerenes. Exohedral fullerenes are formed with substituents outside their cages, while endohedral fullerenes are formed with trapped atomes or molecules inside their cages [27]. These endohedral and exohedral derivatives have been shown to exhibit attractive photonic, electronic, superconducting, lubrication, biomedical, and magnetic properties due to their unique structures [25]. Similar studies have shown that the carbon nanotubes require chemical modifications by attaching the functional groups to improve the compatibility, processing, and solubility with host materials in the engineering of multifunctional materials [29, 30]. Accordingly, the chemical modifications maintain the interesting pristine fullerenes electrochemical, chemical, and physical properties and make them more applicable and reactive for many applications [31].

## 3. Interaction of Fullerene with Aquatic Invertebrates in reference to Bioavailability, Toxicity, and Biosafety

The invertebrates are among the target groups for nanoparticle (NP) ecotoxicity due to their highly developed cellular internalization processes, such as phagocytosis and endocytosis, of nano- and microscale particles that are essential for physiological functions (cellular immunity and intracellular digestion). The biologic, ecologic, and toxicologic characteristics of invertebrates render them a suitable model to detect chemicals and pollutants in typical habitats, primarily via bioaccumulation potential. In addition, invertebrates also offer an advantage to evaluate the individual effect of exposure to the tested chemicals. Assumptions have also been made that the invertebrate model can be used to predict the effects of some toxicants at population and community levels. Thus, they can early indicate the ecosystem function and structure's deterioration or restoration [32]. We are going to discuss the effects of fullerene in aquatic invertebrate organisms in the current section.

First, the chronic toxicity of fullerene C_60_ was tested on *Chironomus riparius*, also known as the harlequin fly, and hypothesized that higher food concentration could reduce the toxic response. This 10-day test was performed by using *Urtica* sp. as food in two different concentrations, which were 0.5 and 0.8%. The test was conducted in sediment that contained fullerene with masses of 0.36 to 0.55 mg/cm^2^. The results demonstrated that at 0.5% food treatment, a significant difference in growth-related endpoints was found, whereas little effect was observed for the higher food concentrations than the control group. Furthermore, although they found agglomerates of fullerene in the gut, the microvilli were damaged. Taken together, this finding demonstrated the fullerene's potential toxicity to *C*. *riparius* in terms of morphological changes and larval growth inhibition [33]. Next, a similar study on the chronic effects of C_60_ on different life stages of *C*. *riparius* in 10-day growth (larvae) and 42-day (adult midges) emergence tests was conducted. The results showed a decrease in body length at a concentration of 0.0025-20 mg/kg C_60_, but effects disappeared at higher concentrations. The study stated that small fullerene agglomerates more significantly affected *C*. *riparius* than the large ones, as shown with high doses of C_60_. Further, a bell-shaped dose-response relationship was observed in the C_60_ exposure results, which might be caused by the relative growth pattern. This dose-response relationship makes ecological risk assessment of C_60_ more difficult since several effects occur at low concentrations [34].

Several prior studies had assessed the toxicity of C_60_ in *Mytilus*. First, the toxicity of C_60_ at 1, 5, and 10 *μ*g/ml concentrations was investigated in marine bivalve *Mytilus*. From the results, the C_60_ expressed no significant effect in lysosomal membrane stability, which indicated an absence of a major toxicity effect. However, C_60_ suspension led to the release of lysozyme and extracellular oxyradical and nitric oxide production in a concentration-dependent manner. Therefore, the results supported the speculation about the bivalve immune system as a key target of NPs [35]. Forward, in a similar study with *Mytilus* sp., a marine mussel, they were exposed for three days to either 0.10-1 mg/l of C_60_ and 32-100 *μ*g/l of polycyclic aromatic hydrocarbon (PAH) fluoranthene or the combination of both compounds. The observed results depicted a concentration-dependent increase in DNA strand breaks caused by C_60_ and fluoranthene individually; however, their combined exposure enhanced the level of DNA strand breaks together with a twofold increment in total glutathione (GSH) content, indicating oxidative stress. The work concluded the generation of toxic response and damage with additive effects. Afterward, the research group suggested further analysis of C_60_ and fluoranthene alone or in combination for a longer duration to establish concrete toxicity results [36]. Further, *Mytilus galloprovincialis Lam*. were also exposed to C_60_ at 0.01, 0.1, and 1 mg/l concentration for 72 h. Later, the accumulation of C_60_ in the digestive gland was demonstrated to induce dephosphorylation of mTOR with no oxidative stress for cellular distribution of C_60_ at 0.01 mg/l concentration. The study suggested that mussels' most affected functions were related to the organization of the cytoskeleton, energy metabolism, and lysosomal activity [36, 37]. Lastly, in another report on *Mytilus galloprovincialis*, the mussels were exposed to C_60_ (0.01, 0.1, and 1 mg/l), B[a]P (5, 50, and 100 *μ*g/l), and B[a]P (5, 50, and 100 *μ*g/l) in combination with C_60_ (1 mg/l). Afterward, the uptake of each treatment group was measured by a different set of chromatography techniques. The study supports the hypothesis of an interaction between these two compounds and demonstrated an antagonistic relationship at the genotoxic and proteome expression level. However, as this effect is observed on a single concentration, the research group further suggested that other dosage concentrations should also be investigated [37, 38].

Further, ecotoxicities of several fullerenes (C_60_, C_70_, and C_60_-phenyl-C_61_-butyric acid methyl ester (PCBM)) on *Lumbriculus variegatus* (California blackworm), a benthic organism, were assessed. The results indicated that 25 to 150 mg/kg of C_60_ reduce the population growth of *L*. *variegatus*, even though no effect on the organism's growth or weight was observed after treated with 25 mg/kg of C_70_ [38, 39]. Similarly, in another study, 10 and 50 mg/kg dry mass of C_60_ were exposed to *L*. *variegatus* for 28 days. Later, it was found that the survival rate or reproduction of *L*. *variegatus* was not affected by C_60_. However, the impairment of feeding activity observed in the study indicated the C_60_'s disruptive effect on worm feeding. In addition, electron and light microscopy also detected C_60_ agglomerates in fecal pellets, and they were not absorbed in gut epithelial cells. This study also reported that through feeding and egestion, fullerene transfer from sediment-to-sediment surface occurred in *L*. *variegatus*. This phenomenon might increase the bioavailability of C_60_ to epibenthic organisms and facilitate the C_60_ transfer in the food web [39, 40].

The acute toxicity of C_60_ was also assessed on *Daphnia magna*, *Hyalella Azteca*, and *Copepods*. The 21-day exposure of C_60_ in different concentrations (2.5 and 5 ppm) generated a significant delay in molting and a reduction in offspring production, producing impacts at a population level [40, 41]. Further toxicity tests were performed in *Daphnia magna* and *Moina macrocopa* with 4 h/d sunlight exposure, testing the photo-toxicity of fullerene by the environmental level of ultraviolet light. The neonates were exposed acutely to aqu/nC_60_ filtered through 0.2 *μ*m (0, 0.462, 0.925, 1.85, 3.70, and 7.40 mg/l) and filtered through 0.45 *μ*m (0, 0.703, 1.40, 2.81, 5.62, and 11.2 mg/l) at 21 ± 1°C, and observations were recorded at 24, 48, 72, and 96 hours. From the results, antioxidant enzyme activities were observed to be increased by coexposure to C_60_ aqueous suspensions and sunlight. The results demonstrated that fullerene led to oxidative damage to *D*. *magna*, aggravated by natural sunlight [41, 42]. Next, in another study, C_60_ NPs were prepared at different concentrations by sonication (0.5, 1, 2, 3, 4, 5, 6, 7, 8, 9, and 10 ppm) and ultrafiltration (40, 180, 260, 350, 440, 510, 700, and 880 ppb) before exposure to *Artemia salina* for 1, 6, 12, 24, 36, 48, and 96 h for acute toxicity testing. The results showed that sonicated C_60_ caused varied mortalities in different chosen stages of this brine shrimp at 15-24 h, 24-48 h, 72-96 h, 6 to 7 days old, and adult. In contrast, filtered solution showed increased mortality with increased C_60_ NP concentrations [42, 43]. Next, a prior study analyzed the antioxidant and oxidative damage responses in the several regions of *Lophiotoma acuta* (marbled turrid) and a bacterium (feeding on mucus produced by *L*. *acuta*) after C_60_ exposure for 24 h. After 1.0 mg of C_60_/l was administered, low levels of antioxidant capacity and lipid peroxidation were displayed in the anterior region of *L*. *acuta*, indicating that complex interactions between estuarine organisms and associated bacteria could be compromised by nanomaterials, which is C_60_ in this case. This finding highlights the importance of proper evaluation of C_60_ usage, considering the ecological consequences [43, 44]. Next, C_60_ was tested in *Daphnia magna* for 48 h acute toxicity tests. From the results, C_60_ increased the mortality rate with an increased concentration of C_60_ and caused even more severe toxicity effects at lower C_60_ concentrations [44, 45]. Similarly, the amount of C_60_ stored in the body at a particular time due to the exposure of C_60_ was also evaluated on *Daphnia*. After being treated with 1 mg/l of C_60_, C_60_ was taken with a body burden of 413 *μ*g/g wet weight. C_60_ was observed to be accumulated significantly in the gut of *Daphnia*. Moreover, gut impairments, reduced digestion and filtration rates, and inhibited several digestive enzymes, such as *β*-galactosidase, trypsin, cellulose, and amylase, were shown after the exposure. The research work provides evidence for limitation in energy acquisition and increases in oxidative damage in *Daphnia* that might be associated with the C_60_ bioaccumulation, which causes immobility and mortality later [45, 46]. In another similar study with the same animal model, the toxicity of C_60_ in artificial freshwater was investigated. After 2 mg/l of fullerene solution was administered for 24 hours, the wet weight of the organism's wet mass was 4.5 ± 0.7 g/kg. However, after depuration for 24 and 48 h in clean water, 46% and 74% of accumulated fullerene, respectively, were eliminated. Also, large aggregates of fullerenes were found in the gut of *Daphnids*. Taken together, the study suggested that *D*. *magna* might have contributed to carrying fullerene from one trophic level to another because of their significant uptake of fullerene and relatively slow depuration [46, 47]. Further, another behavior study in *D*. *magna* also discovered that chronic exposure of nC_60_ (21 days) in different concentrations (0.1 or 1 mg/l) yielded a reduction in their feeding ability, hopping, and heartbeat frequencies. Later, the transcriptome analysis depicted this phenomenon which is possibly due to alterations in some underlying physiological functions, including protein degradation, reproduction, energy metabolism, and cell structure repair [37, 38]. Similarly, C_60_ and PCBM (is a solubilized version of the C60) were not found to be acutely toxic when tested at 5, 10, 25, and 50 mg/l for 21 days, while C_70_ had significant acute toxic effects. However, C_60_, C_70_, and PCBM depicted heart rate elevation over time [47, 48]. In addition, another study demonstrated that accumulated C_60_ in *D*. *magna* could be transferred to zebrafish through dietary exposure and accumulated mostly in the intestines; however, no magnification was found [48, 49].

Taken together, we envisage that when exposed to *Daphnia magna*, the suspended C_60_ nanoparticles revealed protection against short-term UV and fluoranthene photo-induced toxicity, although it caused cellular damage. The cellular components, such as microvilli, mitochondria, and basal unfolding, are protected by C_60_, which evidenced by transmission electron microscopy in organisms after being shortly exposed to UV and fluoranthene photo-toxicity, while longer exposure time (21 days) of C_60_ at low concentration led to significant cellular damage in the alimentary canal of *Daphnia magna* [49, 50]. Further, when C_60_ was analyzed in *Perinereis gualpensis* (a Polychaete species), there were no oxidative damage, GSH (glutathione), and GCL (glutamate cysteine ligase) observed in all tested concentrations, even though after 2 and 7 days, the antioxidant capacity was found to be elevated in the treated group, suggesting a possibility for fullerene acting as an antioxidant [50, 51]. The summary of the toxicity of fullerene-based nanomaterial to aquatic invertebrates is described in Table 1.

## 4. Interaction of Fullerene with Aquatic Vertebrates in reference to Bioavailability, Toxicity, and Biosafety

Identification of nanomaterial toxicity is challenging since the potential usage of nanomaterial exposes them to the environment and eventually harms human health. The assessment of cytotoxicity of nanomaterials is important to understand the actual interaction and interpretation in a biological organism. The quality of NPs depends on the dispersion medium used for their suspension, which may add to their cytotoxicity potential. The main concern of fullerene testing is to identify the associated risk factors. In addition, the defined conditions of laboratories play a large part in understanding the toxicology profile. The comprehension of nanomaterial interaction inside the biological body is essential to understand all aspects of cells, organs, and blood systems. The vertebrate system plays a significant part in providing cheap, easy, and time-efficient animal models to assess toxicity rapidly. In a prior study, fullerenes (100-500 ppb of C_60_ and C_70_ and 500-5000 ppb of C_60_(OH)_24_) were administered 24-96 hours of postfertilization (hpf) zebrafish embryo. The results showed that while C_60_ alone led to apoptotic and necrotic cell death, both C_60_ and C_70_ were demonstrated to cause mortality, malformations, and pericardial edema in the embryos. On the other hand, even though C_60_(OH)_24_ exposure increased embryonic cellular death, it did not lead to apoptosis. Therefore, the study suggested less toxicity of C_60_(OH)_24_ compared to C_60_ [52]. Furthermore, in a similar study in the embryonic zebrafish model conducted by Henry et al., the depletion of C_60_ from exposure medium and embryonic zebrafish uptake was evaluated [53]. Later, it was found that around 90% of C_60_ could be recovered from zebrafish embryo extracts. The toxicological assay revealed that sorption to test vials caused the loss. After 6 hours, this absorption already resulted a decrease of exposure solution to less than 50% of the initial dose. The embryo uptake of C_60_ increased throughout the 12 h exposure. The study suggested that it is necessary to measure the time course of the C_60_ dose to determine the range of concentrations to which the organism will be exposed. Furthermore, in a prior experiment done by Zhu et al., the *Danio rerio* embryo was exposed to nC_60_ to analyze the developmental toxicity in a 96 h exposure [54]. The results demonstrated that 1.5 mg/l of nC_60_ delayed the development of the zebrafish embryo and larvae, reduced the hatching and survival rates, and caused pericardial edema, whereas 50 mg/l of fullerol hydroxylated C_60_ derivative did not affect the zebrafish embryos. The study also showed that the addition of an antioxidant (glutathione) mitigated the toxicity, suggesting the developmental toxicities are regulated by a free radical-induced mechanism or another form of oxidative stress. Furthermore, in the following study reported by Henry et al., C_60_ in two different forms (C_60_-water) and tetrahydrofuran (THF-C_60_) were exposed to larval zebrafish to assess the changes in survival and gene expression [55]. The results demonstrated that the zebrafish larval survivability was compromised in THF-C_60_ and THF-water. However, this phenomenon was not observed in the C_60_-water treatment group. In addition, in terms of gene expression, the biggest difference was displayed in the THF-C_60_ group. The research indicated that toxic effects found in this study might be associated with the products of THF degradation rather than C_60_. Additionally, this also may explain the C_60_ toxicity found in other findings.

In another *in vivo* study conducted by Sarasamma et al. [56], waterborne C_60_ was exposed to adult zebrafish for 12 days at 1 and 2 ppm concentrations, respectively, and fish's behavioral alterations were measured by phenomics approach. The results showed the alteration in fish's locomotor activity, response to a novel environment, aggression level, shoal formation, and color preference. Moreover, the fish also displayed dysregulation in the circadian rhythm locomotor activity. The corroboratory biochemical test results showed the induction of oxidative stress and DNA damage, followed by a significant reduction in antioxidative capacity and ATP level. The research group concluded low concentration of C_60_-induced multiple behavioral abnormalities in adult zebrafish. Similarly, in another prior study also conducted by Sarasamma et al. [57], the potential adverse effects of fullerene C_70_ exposure were assessed on adult zebrafish. Two different doses, 0.5 ppm and 1.5 ppm, were exposed to adult zebrafish for two weeks. Similar to C_60_ results, the results showed abnormalities in locomotion, explorative behavior, aggressiveness level, conspecific social interaction behavior, shoal formation, anxiety elevation, and circadian rhythm locomotor activity. Also, biochemical marker tests revealed a significant increase in superoxide dismutase (SOD), reactive oxygen species (ROS), cortisol, Hif 1-*α*, ssDNA, TNF-*α*, and IL-1*ꞵ* in brain and muscle tissue, concluding several and similar toxic effects in altering the neurobehavior parameters of zebrafish after exposed to fullerene-based nanomaterials [57]. Taken together, the studies performed on *Danio rerio* in the embryos and adult stages indicate the importance of concentration range and exposure time in assessing the toxicity effect of fullerene over vertebrate model since different toxicological effects are observed in different studies under different concentrations and tests performed. To establish a concrete result of the toxicological effect of fullerene, it is necessary to accumulate more data on different parameters.

Next, in a previous study done by Oberdörster, when 0.5 ppm of uncoated nC_60_ was exposed to largemouth bass, *Micropterus salmoides*, for 48 hours, it resulted in significant lipid peroxidation in the brain [58]. In addition, GSH was observed to be marginally decreased in the fish's gills with an increase in water clarity. Further, in another experiment on *Anabas testudineus*, a freshwater fish, C_60_ was demonstrated to induce toxicity, specifically on reproductive parameters, after being exposed for 60 days at 5 mg/l and 10 mg/l. The results showed a reduction in gonadal steroidogenesis with a decrease in steroidogenic enzymes, 3*β*- and 17*β*-hydroxysteroid dehydrogenase. Furthermore, a significant decrement was also observed in the serum testosterone and estradiol in male and female fish, respectively, in concentration- and time-dependent manners. Thus, the study suggested stress induced by administration of C_60_ leads to reproductive toxicity in *Anabas testudineus* [59]. In a similar study protocol on *Anabas testudineus*, the toxic effect of C_60_ was evaluated on their behavior and hematology levels at 5 and 10 mg/l for 96 h and 60 days. The decline in acetylcholinesterase (AChE) enzyme activity in brain tissue showed prominent changes in fish behavior. Furthermore, the hematological parameter showed a significant reduction in blood cells with increased alanine and aspartate aminotransferase in serum. The results concluded that the sublethal concentration of C_60_ generates toxicity by affecting the normal physiology of *A*. *testudineus*, which might affect the ecosystem's health status [59, 60].

Forward, in another prior study by Sumi and Chitra and Blickley and McClellan-Green., the role of C_60_ was evaluated on the role of the brain antioxidant system of cichlid fish, *Pseudetroplus maculatus* [60, 61]. In their study, 0.1 mg/l of C_60_ was administered to the fish for 96 hours. The results demonstrated no significant alterations in terms of the brain weight, whereas the notable reduction in antioxidant enzymes (like catalase, SOD, and GSH) and a significant increase in hydrogen peroxide and lipid peroxidation were found after 48 h C_60_ treatment. Also, acetylcholinesterase (AChE), a marker enzyme for the brain, showed a significant reduction after exposure to C_60_ at the end of 48-96 h. Thus, the work showed that the administration of C_60_ has adverse effects on the fish brain. The aqueous suspensions of C_60_ aggregates were studied on marine teleost *Fundulus heteroclitus* of an embryo, larvae, and adult stages. In natural seawater, the aggregates of C_60_ are mixed and precipitated in bottom water after 24 hours, resulting in very low mortality. No median lethal doses could be calculated at a concentration below 10 mg/l. In addition, even though the C_60_ aggregates were attached to chorion, no effect in the development of the embryos on hatching success was observed. With higher exposure levels, the movement of C_60_ from chorion into the embryo tended to increase together with a dose-dependent increase in GSH and a decrease in lipid peroxidation (LPO) [61]. Further, the C_60_ exposed to *Cyprinus carpio* (common carp) demonstrated no effect on viability, whereas hampered growth occurred after 3 h of exposure to several concentrations of C_60_, which were 0.1, 1, and 10 mg/l. Also, higher antioxidant competence to peroxyl radicals was observed in this fullerene than in other reactive colonies [62, 63].

On the contrary, another study exposed three different chemical toxins, which were 6-hydroxydopamine, gentamicin, and cisplatinum, to a whole animal system in order to investigate the fullerenes' ability to protect the animal from the toxins. This model is useful to predict the toxicity and efficacy of this fullerene in mammals. When water-soluble fullerenes in both positive and negative charges were exposed to the zebrafish embryos at 1 and 500 *μ*M for 24-120 hpf, the results indicated that the fullerenes could give protection against the toxins, which can induce apoptotic cell death in a vertebrate. Furthermore, this work suggested that the relative potential for these compounds' pharmacologic use varies significantly with respect to stability [63, 64]. Hence, more studies on different parameters of concentration, time, environmental factors, and vertebrate model are necessary to understand the toxic as well as beneficial properties of fullerene nanomaterials. We have compiled the results of different toxicity studies in Table 2.

## 5. Biodistribution and Fate of Fullerene after Ingestion by Aquatic Organisms

The biodistribution of fullerene on ingestion by aquatic organisms has been seldom addressed in the literature. Moreover, the concentration of fullerenes in many environmental matrices is still unknown, while quantification methods are under development [33, 65, 66]. However, NPs within cells may cause alterations in the cytoskeletal network [67, 68]. Waissi-Leinonen and colleagues depicted the agglomeration of fullerene C_60_ in the gut area and damaged the microvilli of *C*. *riparius* [33]. Furthermore, in another study by Sforzini et al. and Barranger et al., C_60_ was reported to be distributed in mussel digestive gland cells [36, 37]. In addition, the inhibition of mTOR might also be involved in pathophysiological perturbation induced by nanoparticle accumulation. Further, they stated that autophagic induction by fullerene C_60_ might reflect the degradation of unrecognized materials attempted by lysosomes. These materials might be identified by cells as damaged intracellular proteins and membranes or pathogens. The statement was made based on observations, where C_60_ accumulation in the lysosomal-vacuolar system of the epithelial cells is in the major digestive gland. Overall, the report suggested that dysregulation of mTOR 1 and 2 might inhibit the growth and reproduction of cells and organisms. Also, in a study, Pakarinen et al. and Oberdörster et al. reported that fullerene concentration in feces of *L*. *variegatus* was high in comparison to the bulk sediments [39, 40]. The study suggested that high fullerene concentration in the form of pellets might partially stem from worm's consumption and absorption of some sediment fraction for nutritional purposes, whereas the fullerenes and other particles are excreted. Further, Tao et.al and Rouse et al. exposed *D*. *magna* to 0, 0.01, 0.02, 0.04, 0.06, and 1.0 mg/l for seven days and the results showed an increment in body burden of C_60_ along with a higher dose and bigger particle size [69, 70]. Thus, the various papers and results suggest that more research is needed to understand the risks that fullerene may pose in sediments and organisms' bodies in different doses and concentrations under specific environmental conditions. Therefore, the limited information in the fullerene biodistribution is an important topic for future research.

## 6. Discussion Based on Current Understanding

The manufacturing of fullerenes to fit in for a specific task demands changes in their surface chemistries and properties. With the improvement according to demand, the fullerenes acquire novel physicochemical properties to be assessed for potential toxicological behavior compared to the natural ones. The impact of fullerenes via direct contact with water containing the amount of fullerene through skin and inhalation and via an indirect consumption of aquatic organisms exposure might pose a serious threat to human health in the long run [65, 67, 71, 72]. Until now, the real effects associated with the interaction of fullerenes with the aquatic organisms are still lacking and remain challenging to analyze in the absence of relevant data.

The methods to evaluate the toxicity of fullerenes are evolving in recent years. The current understanding of the toxicity of fullerene must acknowledge that data compilation limitation serves as a barrier to understanding the interaction of fullerene inside the organism's body to interpret the results firmly. In reviewing the emerging environmental problem, it is highlighted that all significant effects on environmental fate, transport, and bioavailability of cocontaminants play a crucial part in understanding fullerene's toxicology.

The toxicity of fullerenes is, to date, poorly understood and contradictory in some cases. However, experimentation on fullerene toxicity testing has demonstrated that fullerene is toxic in some forms. Studies have shown that ROS and free radical production are among the main mechanisms of nanotoxicity; in turn, they might lead to inflammation, oxidative stress, and consequent damage to proteins, membranes, and DNA [69, 73]. In a study, Oberdörster showed that fullerenes caused damage in fish brains [58]. Also, Fortner et al. and Howard demonstrated that fullerenes killed water fleas and showed bactericidal properties [66, 71]. Furthermore, Sayes et al. and Daughton stated that toxicity is a sensitive function of surface derivatization [68, 72], while Rouse et al. and Nel et al. reported that the extent of aggregation, emulsion bases, and different solvents are important variables in the formation of aggregates [70, 73].

The studies discussed here with fullerene toxicity response on invertebrate and vertebrate models depicted contradictory results. Although fullerene C_60_ has been shown to cause mortality, ROS production, aggregation, and lipid peroxidation on a large basis, some studies have reported conflicting results. Waissi-Leinonen et al. demonstrated the effect of C_60_ in relative growth patterns of *C*. *riparius* in a bell-shaped dose-response manner [34]. Rajasree et al. and Marques et al. showed that exposure of sonicated C_60_ resulted in varied mortality in different stages of *A*. *salina*, whereas the filtered solution of C_60_ upon exposure revealed increasing mortality with an increase in concentration [42, 43]. Similarly, Blickley and McClellan-Green and Letts et al. also suggested the nontoxic effect of water-stirred suspensions of C_60_ at a concentration up to 10 mg/l in *H*. *heteroclitus* at different life stages [61, 62].

Next, in another study, Tervonen et al. and Wang et al. mentioned the role of *D*. *magna* in carrying fullerene from one trophic level to another [46, 47]. Similarly, Pakarinen et al. and Oberdörster et al. demonstrated the fullerenes transfer process from sediment to sediment surface by *L*. *variegatus* through feeding and egestion [39, 40]. This observation indicated that this transfer might potentially increase the fullerenes' bioavailability to epibenthic organisms, which might be more sensitive to the exposures and might further assist the transfer process in the food chain. Beuerle et al. and Sumi and Chitra stated that positively charged fullerene exhibited greater toxicity in the vertebrate model compared to negatively charged fullerene, which showed a dependency behavior on the structural features [63, 64].

## 7. Conclusions of Future Directions

Taken together, the toxicity of fullerenes to aquatic animals was clearly demonstrated. Since it has great medical implication potentials, it should be studied further in the future. We propose that techniques such as behavioral assay by phenomics, tissue distribution analysis by MASS spectrum, or isotope labeling for *in vivo* tracking should be used to study fullerene-induced toxicity in aquatic animals (i.e., specific biomarkers study, biodistribution, and interaction within an organism's body) to accumulate relevant data required to achieve a clear picture about fullerene-induced toxicity.

## Figures and Tables

**Table 1:** Fullerene-based nanomaterial toxicity in aquatic invertebrates.

Fullerene	Model organism	Dosage and time	Toxic effect	LC50	Reference
C_60_	*Chironomus riparius*	10 g wet artificial sediment and 40 ml C_60_, food source 0.5 and 0.8% *Urtica* sp. 10 days	Morphological changes and inhibiting larval growth. Agglomeration in gut and damage of microvilli.	NA	[33]
C_60_	*Chironomus riparius*	Artificial sediment 0.0004-80 mg/kg dry weight 10 days and 42 days	C_60_ resulted in a bell-shaped dose-response relationship in view of the relative growth patterns.	NA	[34]
C_60_	*Mytilus galloprovincialis Lam*.	1, 5, and 10 ppm 30 minutes to 4 hours	Concentration-dependent lysozyme release, extracellular oxyradical, and nitric oxide production.	NA	[35]
C_60_	*Mytilus galloprovincialis Lam*.	0.01, 0.1, and 1 ppm 72 h	C_60_ accumulated in the digestive gland-induced dephosphorylation of mTOR.	NA	[36, 37]
C_60_ and fluoranthene alone and combination	*Mytilus sp*.	0.10–1 ppm 32–100 ppb 3 days	C_60_ and fluoranthene evoke toxic responses and genetic damage. The combined exposure produced enhanced damage with additive rather than synergistic effects.	NA	[36, 51]
C_60_, C_70_, and C_60_-PCBM	*Lumbriculus variegatus*	0, 10, 25, 100, 150 ppm 28 days	C_60_ can affect the population growth of *L*. *variegatus* but C_60_-PCBM and C_70_ effects are lower in comparison.	NA	[38, 39]
C_60_	*Lumbriculus variegatus*	10 and 50 ppm 28 days	Impairment of feeding activity and C_60_ aggregate presence in feces.	NA	[39, 40]
C_60_	*Daphnia magna, Hyalella azteca, copepods*	30 ppm 5 days 7 ppm 48-96 h 0, 3.75, 7.5, 15, and 22.5 ppm 96 h	C_60_ 21-day *Daphnia* exposure resulted in a significant delay in molting and reduced offspring production at 2.5 and 5 ppm.	NA	[40, 41]
C_60_	*Daphnia magna* and *Moina macrocopa*	4 hr/d sunlight C_60_ filtered 0.2 *μ*m (0, 0.462, 0.925, 1.85, 3.70, and 7.40 ppm) and 0.45 *μ*m (0, 0.703, 1.40, 2.81, 5.62, and 11.2 ppm) 24, 48, 72, and 96 h	Fullerene leads to oxidative damage to *D*. *magna* and it was aggravated by natural sunlight.	0.2 *μ*m—96 h LC50—5.95 ppm 0.45 *μ*m—96 h LC50; >11.2 ppm (outdoor) 0.2 *μ*m—96 h LC50 1.35 ppm 0.45 *μ*m—96 h LC50 1.58 ppm (outdoor)	[41, 42]
C_60_	*Artemia salina*	Filtered C_60_ 40, 180, 260, 350, 440, 510, 700, and 880 ppb Sonicated C_60_ 0.5, 1, 2, 3, 4, 5, 6, 7, 8, 9, and 10 ppm for 1, 6, 12, 24, 36, 48, and 96 h	Exposure to sonicated nanoparticles shows varied mortalities in different stages of *A*. *salina*, whereas filtered solutions showed increased mortality with the increase in concentration.	Sonicated C_60,_ the adult LC50 value was 3.17 ppm, whereas it was 617 ppb for the filtered solution.	[42, 43]
C_60_	*Laeonereis acuta*	0.01, 0.10, or 1.00 ppm 24 h	*L*.*acuta* anterior region presented lower antioxidant capacity and lipid peroxidation after exposure to 1.0 mg C_60_/l.	NA	[43, 44]
C_60_	*Daphnia magna*	Filtered C_60_ 40, 180, 260, 350, 440, 510, 700, and 880 ppb Sonicated C_60_ 0.2, 0.45, 0.9, 2.25, 4.5, 5.4, 7.2, and 9 ppm 48 h	C_60_ caused an increase in mortality with an increase in concentration and higher levels of toxicity at lower concentrations.	Filtered C_60_ 460 ppb Sonicated C_60_ 7.9 ppm	[44, 45]
C_60_	*Daphnia magna*	1, 5, 10, 20, and 40 ppm 72 h	C_60_ exposure restricted-energy acquisition and induced oxidative damage, which might be the mechanisms underlying the observed acute toxicity of C_60_ to daphnia.	16.3 ± 0.8 ppm	[45, 46]
C_60_	*Daphnia magna*	Accumulation 0, 0.2, 2, 7, 15, 30, and 50 ppm 24 h Depuration 48 h	*D*. *magna* may play a role as a carrier of fullerenes from one trophic level to another.	NA	[46, 47]
C_60_	*Daphnia magna*	Short term 22 ppm and long term 1 ppm 10 and 21 days	C_60_ protected cellular components in organisms exposed to UV and fluoranthene photo-toxicity in short-term exposure, whereas long-term exposure (21 days) of low-level C_60_ caused significant cellular damage in *Daphnia magna* alimentary canal.	NA	[49, 50]
C_60_	*Perinereis gualpensis*	200 g 14 days	The data indicated an absence of toxic responses mediated by oxidative stress in estuarine worms exposed to C_60_ mixed in sediments.	NA	[50, 51]

NA: not available.

**Table 2:** Fullerene-based nanomaterial toxicity in aquatic vertebrates.

Fullerene	Model organisms	Dosage and time	Toxic effect	LC50	Reference
C_60_, C_70_, C_60_(OH)_24_	*Danio rerio* (embryo)	100 and 500 ppb for C_60_ and C_70_ and 500 to 5000 ppb for C_60_(OH)_24_	Exposure to C_60_ induced both necrotic and apoptotic cell deaths in the embryo, while C_60_(OH)_24_ induced an increase in embryonic cellular death. Results obtained suggest C_60_(OH)_24_ is significantly less toxic than C_60_.	C_60_/C_70_—200 ppb C_60_(OH)_24_—4000 ppb	[52]
C_60_	*Danio rerio* (embryo)	100, 200, and 400 ppb 2, 6, 12 h	Concentrations of C_60_ decreased to levels not associated with mortality, <50 *μ*g/l, 100% mortality results when the embryos were exposed to concentrations from 250 to 130 ppb.	130 ppb	[53]
nC_60_, fullerol	*Danio rerio* (embryos)	nC_60_—1.5 ppm C_60_(OH)_16–18_—50 ppm 96 hpf	nC_60_ at 1.5 ppm delayed the zebrafish embryo and larval development, decreased the survival and hatching rates, and caused pericardial edema, whereas fullerol hydroxylated C_60_ derivative at 50 ppm did not exert to the zebrafish embryos.	NA	[54]
Water-soluble fullerenes (1-12)	*Danio rerio* (embryos)	1, 10, 100, and 250 *μ*M for 24 hours	Positively charged water-soluble fullerenes tend to exhibit greater toxicity than negatively charged fullerenes with similar structures; toxicity varies considerably among negatively charged fullerenes from very low to moderate, depending on structural features.	Cationic fullerenes—120 *μ*M Anionic fullerenes—500 *μ*M	[63, 64]
C_60_	*Danio rerio* (adult)	1 and 2 ppm for 1 day	C_60_ exposure to adult zebrafish at low concentration induces multiple behavioral abnormalities.	NA	[56]
C_70_	*Danio rerio* (adult)	0.5 and 1.5 ppm for 2 weeks	Toxicity and the alterations were observed in several neurobehavior parameters after zebrafish exposure to environmentally relevant amounts of C_70_.	NA	[57]
nC_60_	*Micropterus salmoides* (juveniles)	0.5 ppm and 1 ppm for 48 h	Increase in lipid peroxidation in the brains at 0.5 ppm and marginal depletion of glutathione (GSH) in the gills.	NA	[58]
C_60_	*Anabas testudineus*	5 and 10 ppm for 60 days	Stress induced by fullerene C_60_ exposure provoked reproductive toxicity in the fish, *Anabas testudineus*.	96 h LC50—50 ppm	[59, 64]
C_60_	*Anabas testudineus*	5 and 10 ppm for 96 h and 60 days	Sublethal concentrations of fullerene C_60_ have a toxic impact on fish *A*. *testudineus* by affecting normal physiology.	96 h LC50—50 ppm	[59, 60]
C_60_	*Pseudetroplus maculatus*	0.1 ppm for 96 h	Decrease in SOD, CAT, GSH reductase, AChE. Increase in hydrogen peroxide and lipid peroxidation.	NA	[60, 61]
C_60_	*Fundulus heteroclitus*	0, 1, 2.5, and 10 ppm for 96 h	Water-stirred suspensions of nC_60_ are not toxic to embryonic, larval, or adult stages of *F*. *heteroclitus* at concentrations up to 10 ppm.	NA	[61, 62]
C_60_	*Cyprinus carpio*	0.1, 1, and 10 ppm for 48 h	The results indicated that C_60_ affects bacterial communities that live in mucus secretions of common carp.	NA	[62, 63]

NA: not available.

## Data Availability

Data is available upon request to authors.
